# Tellurium doped zinc imidazole framework (Te@ZIF-8) for quantitative determination of hydrogen peroxide from serum of pancreatic cancer patients

**DOI:** 10.1038/s41598-020-78115-6

**Published:** 2020-12-03

**Authors:** Batool Fatima, Dilshad Hussain, Adeela Saeed, Muhammad Salman Sajid, Saadat Majeed, Rahat Nawaz, Muhammad Naeem Ashiq, Muhammad Najam-ul-Haq, Rubaida Mehmood

**Affiliations:** 1 Department of Biochemistry, Bahauddin Zakariya University, Multan, 60800 Pakistan; 2 HEJ Research Institute of Chemistry, International Centre for Chemical and Biological Sciences, University of Karachi, Karachi, 75270 Pakistan; 3 Department of Chemistry, The Women University Multan, Multan, Pakistan; 4 Institute of Chemical Sciences, Bahauddin Zakariya University, Multan, 60800 Pakistan; 5 MINAR Cancer Hospital, Pakistan Atomic Energy Commission, Islamabad, Pakistan

**Keywords:** Biochemistry, Biomarkers, Chemistry, Materials science, Nanoscience and technology

## Abstract

The tellurium doped zinc imidazole framework (Te@ZIF-8) is prepared by a two-step hydrothermal strategy for the electrochemical sensing of hydrogen peroxide. Material is characterized by transmission electron microscopy (TEM), energy-dispersive X-ray spectroscopy (EDS), X-ray diffraction (XRD), thermogravimetric analysis (TGA), and differential scanning calorimetry (DSC). The electrochemical characterization of the MOF modified electrode is done by a three-electrode system. Electrochemical sensing of hydrogen peroxide is made by cyclic voltammetry, amperometry, and impedance measurements. Results demonstrate that Te@ZIF-8 shows a detection limit of 60 µM with linearity up to 0.98855. Material is stable to 1000 cycles with no significant change in electrochemical response. Amperometry depicts the recovery of hydrogen peroxide from human serum up to 101%. Impedance curve reveals the surface of Te@ZIF-8-GCE (glassy carbon electrode) as porous and rough and an interface is developed between analyte ions and the sensing material. Finally, the modified electrode is used for the quantitative determination of hydrogen peroxide from serum samples of pancreatic cancer patients, diagnosed with CA 19-9.

## Introduction

Cancer is a leading cause of death worldwide. Cancer cells have abnormalities not only in their genetic sequences but also in their patterns of gene expression, and uncontrolled proliferation is the hallmark of cancer cells. Pancreatic cancer has become a major clinical challenge and one of the dangerous malignancies^1,2^. It has been ranked as the fourth main cause of death because of its vigorous nature, late clinical diagnosis, and limited treatment procedures^3^. Researchers have found several clinically important biomarkers for earlier detection. The miRNAs are critical post-translational regulators of gene expression and are small non-coding RNAs with endogenous expression^4^. For the clinical detection of cancers such as breast, non-small-cell lung, colon, ovarian, prostate, and pancreatic, the abnormal expressions of these miRNAs function as a prognostic marker^5,6^. Antigen like CA 19-9 is highly expressed during the conditions of pancreatic cancer.

Molecular oxygen is comparatively less reactive, however, oxygen derivatives are highly reactive that are involved in various chemical reactions and are produced during aerobic metabolism. Reactive oxygen species (ROS) are thus continuously formed in aerobic organisms and more than 150 disorders in humans including cancers are caused by ROS^7^. Normal cells become malignant due to the effect of ROS on DNA^8,9^. ROS such as HYDROGEN PEROXIDE is one of the causes of pancreatic cancer. Due to their oxidative nature, ROS formed by some anti-cancer agents and radiations can kill cancer cells^10,11^. Insufficient amounts, ROS act as a second messenger in the up-regulation of transcription of various proteins and signal transduction^12–14^. So, ROS can affect tumor biology by killing cells, including damage to DNA and up-regulation of molecular expressions. ROS are produced in tumor tissues by infiltrating inflammatory and cancer cells^15–18^. Cancer cells inter-relate with blood and immune cells after release from a primary tumor which produces more ROS. Both elevated as well as decreased levels of ROS promote tumor growth and metastasis.

Hydrogen peroxide is a common but prominent member of the ROS family. Numerous methods have been reported for the detection of hydrogen peroxide including chemiluminescence, fluorescence, spectrometry, electron spin resonance, and electrochemical methods^19–21^. Electrochemical methods are in focus due to their versatility, reproducibility, low cost, and ease of operation. Different modes of electrochemical methods offer reproducibility, selectivity, and sensitivity^22,23^. Several materials including carbon-based^24^, metal/metal oxides^25^, composites, alloys^26,27^, polymers^28^, and metal–organic frameworks^29^ have been used for hydrogen peroxide sensing.

Metal–organic frameworks (MOFs) have been unique because of high surface area, tunable surface, presence of metal inside the structure, and organized 3D geometry^30–32^. Moreover, MOFs have rich surface chemistry for the functionalization of other materials. Owing to these attributes, MOFs are used in almost every field of research including biomedical research^33^, environmental analysis^34^, food science^35^, energy applications^36^, catalysis^37^, separation science^38^, and sensing^39^. In recent years, applications of MOFs in sensing and biomedical research are well-explored^40^. Iron-containing MOFs (MIL-88A) is used for the sensing of single-stranded DNA (ssDNA)^41^, smartphone-assisted MOFs-based biosensor is used for the colorimetric sensing of endogenous biomolecules^42^ and graphene-MOF composites are used for the electrochemical sensing of different biomolecules^43^.

Herein, tellurium doped zinc imidazole framework (Te@ZIF-8) is employed as a catalyst by depositing on glassy carbon electrode for the electrochemical sensing of hydrogen peroxide from biological samples. The effect of ROS particularly hydrogen peroxide is evaluated on pancreatic cancer. Elevated levels of hydrogen peroxide lead to higher expression of CA 19-9, antigen responsible for pancreatic cancer.

## Experimental

### Synthesis of tellurium nanowires

Tellurium nanowires were prepared by the reported method^44^. 2.0 g PVP (polyvinyl pyrrolidone) and 184.3 mg sodium tellurite were added to 64 mL deionized water followed by 3.4 mL hydrazine hydrate and 6.7 mL aqueous ammonia solution with magnetic stirring for 30 min at room temperature. The content was poured into 100 mL Teflon-lined stainless steel autoclave and placed in an oven at 180 °C for 4 h. After cooling, the precipitates were washed by acetone and dispersed in 50 mL methanol.

### Synthesis of Te@ZIF-8

50 mL Zn(NO_3_)_2_·6H_2_O methanolic solution (0.1 mol L^−1^) was added to 50 mL methanolic solution of Te nanowires, followed by the addition of 50 mL 2-methylimidazole (MeIM) methanolic solution (0.8 mol L^−1^) and heated for 2 h. The mixture was centrifuged and washed with an excess of methanol. The product was obtained after freeze-drying. For further pyrolysis, the prepared Te@ZIF-8 was transferred to a ceramic boat and heated at 800 °C for 6 h in an electrical furnace at a heating rate of 5 °C min^−1^. Pure ZIF-8 was synthesized in the same way without tellurium.

### Ethics

All the experiments and studies on serum samples of Pancreatic Cancer patients were carried out according to guidelines and approval of the Ethical Committee of MINAR Cancer Hospital Multan, Pakistan Atomic Energy Commission, Pakistan. All the protocols and experiments were approved by the Ethical Committee.

Informed consent was obtained from all the patients for serum samples used in this study.

### Determination of CA 19-9 levels in serum samples

Elecsys CA 19-9 is a specific immunoassay for the quantitative determination of CA 19-9 in human serum and plasma. CA 19-9 is considered a biomarker for pancreatic cancer. During 1st incubation, a sandwich complex was formed by monoclonal CA 19-9-specific antibody labeled with ruthenium complex and biotinylated monoclonal CA 19-9-specific antibody when 10 μL of the sample was taken. In 2nd incubation, the complex was bound to the solid phase of biotin and streptavidin on the addition of streptavidin-coated microparticles. The time required was 18 min for the whole process. Detailed procedure is given below;

Blood samples of 7 pancreatic cancer patients were collected in commercial sample collection tubes with prior consent. Serum was separated from blood by centrifugation at 14,000*g* at 4 °C. The obtained serum samples were stored at − 20 °C for further analysis. For electrochemical detection of hydrogen peroxide from serum samples, serum samples were diluted twenty times with PBS (pH = 7.4). For the detection of CA 19-9, the Sandwich Elisa principle was applied. A 10 μL serum sample was taken for the 1st incubation. 3 mg L^−1^ biotinylated monoclonal CA 19-9 specific antibody (mouse) preserved in 100 mmol L^−1^ phosphate buffer made sandwich complex with monoclonal CA 19-9 specific antibody (mouse) labeled as 4 mg L^−1^ preserved in 100 mmol L^−1^ phosphate buffer ruthenium complex at pH 6.5. In 2nd incubation, 0.72 mg mL^−1^ streptavidin-coated microparticles were added to the complex. After addition, the complex was bound to the solid phase via biotin and streptavidin. The mixture of microparticles attached to the solid phase was aspirated into a measuring cell, where microparticles were captured magnetically onto the electrode surface. ProCell/ProCell M was used to remove un-attached substances. Chemiluminescent emission took place upon applying the voltage to the electrode that was measured by a photomultiplier. The calibration curve was drawn to check the results generated specifically by the two-point calibration and a master curve provided through the reagent barcode or e-barcode.

### Electrochemical analysis of hydrogen peroxide by Te@ZIF-8-GCE

Hydrogen peroxide was detected by cyclic voltammetry. Electrode conductivity was checked in 0.1 M potassium ferrocyanide containing 0.1 M KCl solution with three electrodes system. Ag/AgCl as the reference electrode, Pt wire as a counter electrode, and glassy carbon with Te@ZIF-8 as working electrode. The measurements were made at room temperature. Experimental parameters like the scan rate, pH effect, and concentration were optimized. Recovery of hydrogen peroxide and analyses from CA 19-9 were amperometrically determined.

## Results and discussion

### Characterization of Te@ZIF-8

**Figure 1:**
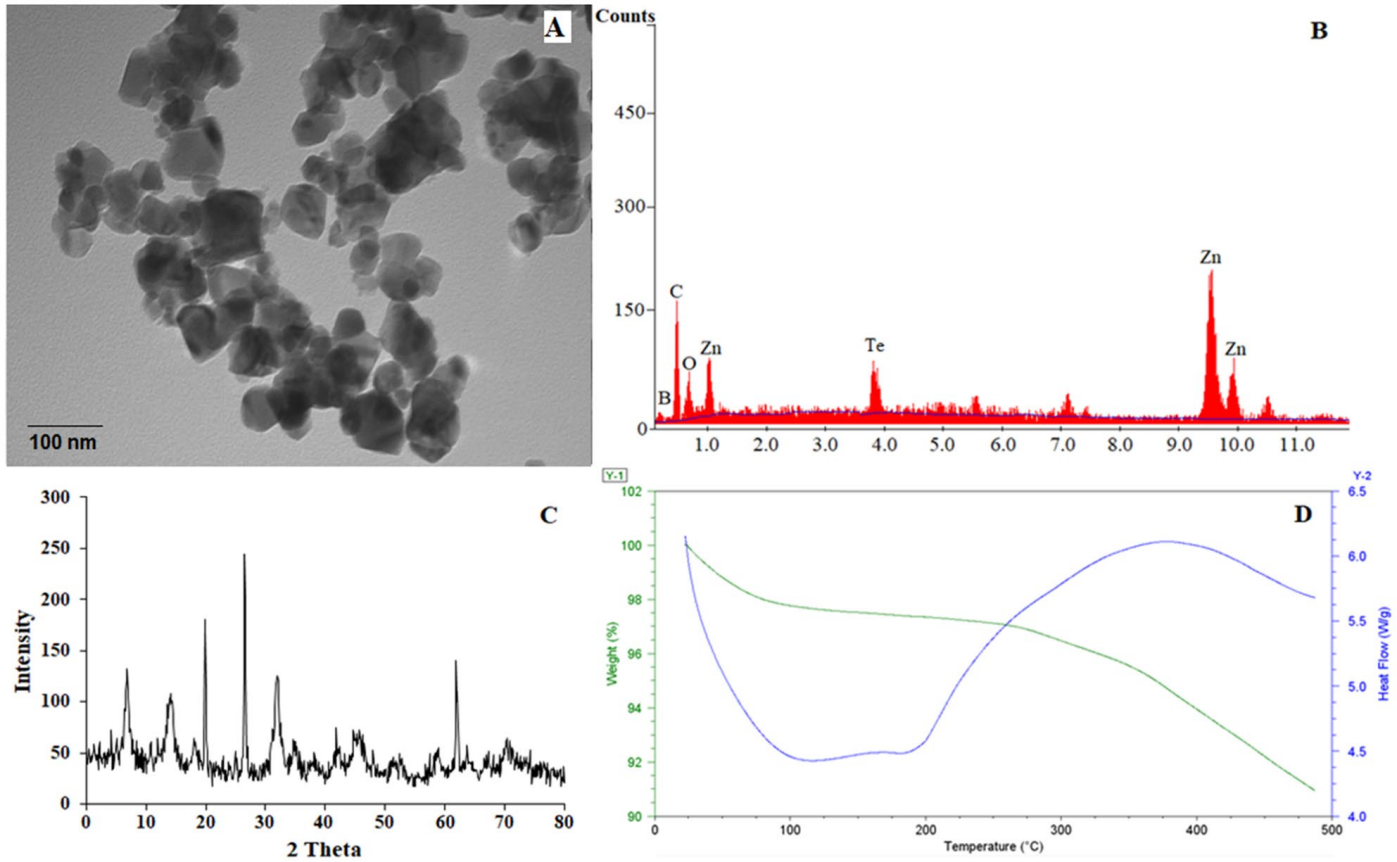
Characterization of Te@ZIF-8 (**A**) TEM, (**B**) EDS, (**C**) XRD, and (**D**) TGA analysis.

Te@ZIF-8 is characterized by transmission electron microscopy (TEM), energy-dispersive X-ray spectroscopy (EDS), X-ray diffraction (XRD), thermogravimetric (TGA) analysis, and differential scanning calorimetry (DSC). The TEM image of MOF (ZIF-8) shows hexagonal structures with regular geometry (Fig. 1A). Approximate particle size is in the range of 60–70 nm. Particles seem attached, attributed to the presence of Te nanowires to which ZIF-8 particles are attached. Te wires are prepared and incorporated into MOF structure via post-synthetic modification. The presence of Te amongst ZIF-8 particles is indicated in EDS analysis through the presence of zinc, carbon, oxygen, and tellurium at their representative positions (Fig. 1B). XRD analysis verifies the formation and phase of Te@MOF (Fig. 1C). Representative peaks of ZIF-8 are at 7°, 13°, 18°, 20°, 27°, 31° and 34°. The simulation pattern also confirms the crystalline hexagonal cubic-phase structure of ZIF-8. TGA and DSC curves of Te@ZIF-8 indicate the thermal response (Fig. 1D). The first curve at 100 °C is attributed to moisture, the second change is observed around 250 °C to 350 °C indicates the decomposition of MOF structure.

### Impedance measurements

**Figure 2:**
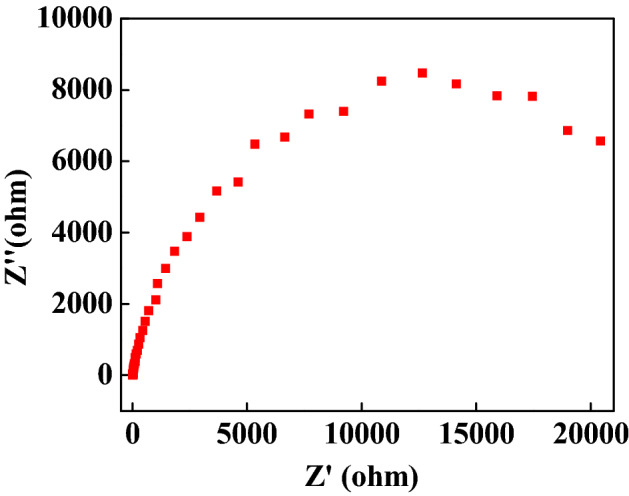
Impedance response of hydrogen peroxide on Te@ZIF-8-GCE.

Impedance studies are based on resistive or capacitive transduction of the sensors and are carried out on Te@ZIF-8-GCE for hydrogen peroxide sensing (Fig. 2). This is the Nyquist plot between the real part (X-axis) and the imaginary part (Y-axis) of the impedance. Each point of coordinates corresponds to the impedance vector value at a different frequency. The obtained value of capacitance is the function of the concentration of bias voltage, the area of the electrode, and the ions. 0.1–0.4 pF/μm^2^ is the specific capacitance for PBS (standard physiological buffer solution). Results reveal the surface of the electrode as rough and porous which leads to the distribution of local time constants and combines to give a global pseudocapacitive behavior. As the duration of applied sinusoid increases, more reagents reach the interface increasing impedance.

### Cyclic voltammetry analysis of hydrogen peroxide by Te@ZIF-8-GCE

Cyclic voltammetry characterization of the prepared catalyst is shown in Fig. S1. Blueline is the conductivity of bare glassy carbon electrode in potassium ferrocyanide and no oxidation and reduction peaks are observed. The black curve is showing the voltammetric response of Te@ZIF-8-GCE towards potassium ferrocyanide. A well defined broad oxidation peak is obtained at the potential region of + 0.1 to + 0.4 V and a reduction peak at 0.0 to + 0.2 V. Te@ZIF-8-GCE conductive response is extended to the sensing of hydrogen peroxide. Reduction and oxidation curves explain the redox activity of electrode material in 0.1 M PBS (pH 7) containing hydrogen peroxide. The mechanism is explained by the following equations;

$${\text{T}}_{\text{e}}+2{\text{H}}_{2}\text{O}\to {\text{TeO}}_{2}+4{\text{H}}^{+}+4{\text{e}}^{-}\;\; (\text{Oxidation})$$

$${\text{T}}_{{\text{e}}} {\text{O}}_{2} + 2{\text{H}}_{2} {\text{O}}_{2} \to {\text{T}}_{{\text{e}}} + 2{\text{H}}_{2} {\text{O}} + 2{\text{O}}_{2} \;({\text{Reduction}})$$

**Figure 3:**
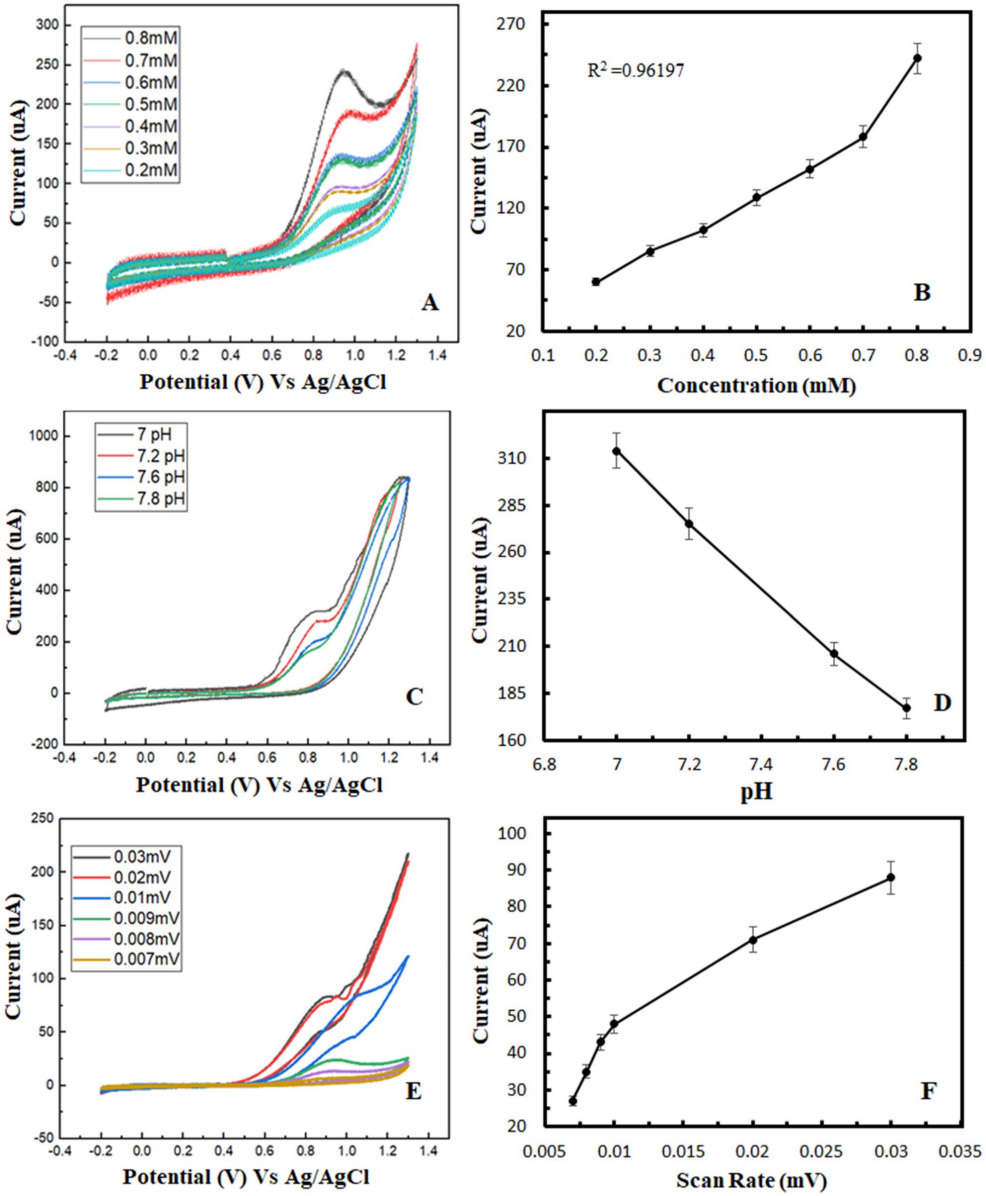
Cyclic voltammograms showing the effect of (**A**) concentration, (**C**) pH, and (**E**) scan rate, and their calibration curves (**B**,**D**, and **F** respectively) on Te@ZIF-8-GCE for the detection of hydrogen peroxide at a potential window of − 0.4 V to + 1.4 V.

CV results for the concentration effect of hydrogen peroxide on Te@ZIF-8 GCE are recorded at concentrations from 0.2 to 0.8 mM (Fig. 3A). The oxidation peak current increases by increasing analyte concentration. Maximum current is noted at 0.8 mM concentration while least oxidation peak current is at 0.2 mM hydrogen peroxide concentration in 0.1 M PBS (pH 7). Figure 3B indicates a direct relation between hydrogen peroxide concentration and the current. For hydrogen peroxide sensing, the modified glassy carbon electrode has a linear dependency of R^2^ = 0.96197.

CV is applied under different pH conditions (7, 7.2, 7.6, and 7.8) to check the effect of pH change on the sensing of hydrogen peroxide by electrode material (Fig. 3C). Current decreases with the pH increase from 7 to 7.8. The calibration curve shows a minimum current at pH 7.8 (Fig. 3D). So, pH 7 is chosen to sense hydrogen peroxide.

Scan rate optimizations are made at scan rates from 0.007 to 0.03 mV/s in 0.1 M PBS of pH 7 (Fig. 3E,F). Current increases constantly by increasing the scan rate. The minimum current is noted at 0.007 mV/s and maximum at 0.03 mV/s, showing a linear relationship between current and scan rate. There is also a direct relation between produced current and potential because of oxidation at different scan rates. The maximum oxidative current is observed at 0.03 mV/s and the R^2^ value for oxidation is 0.9363.

### Interferences studies

The effect of interferences is checked by the addition of interfering species like ascorbic acid and dopamine in a 0.1 mM hydrogen peroxide solution. They are added first in the same concentration as of hydrogen peroxide and then the concentration of dopamine is doubled. Oxidation peak due to hydrogen peroxide becomes prominent by adding interfering agents and reduction peak also appears (Fig. S2). Similarly, a higher current response is observed after the addition of interfering species. The observed peaks are also different from the original peaks observed for dopamine and ascorbic acid.

### Stability studies

**Figure 4:**
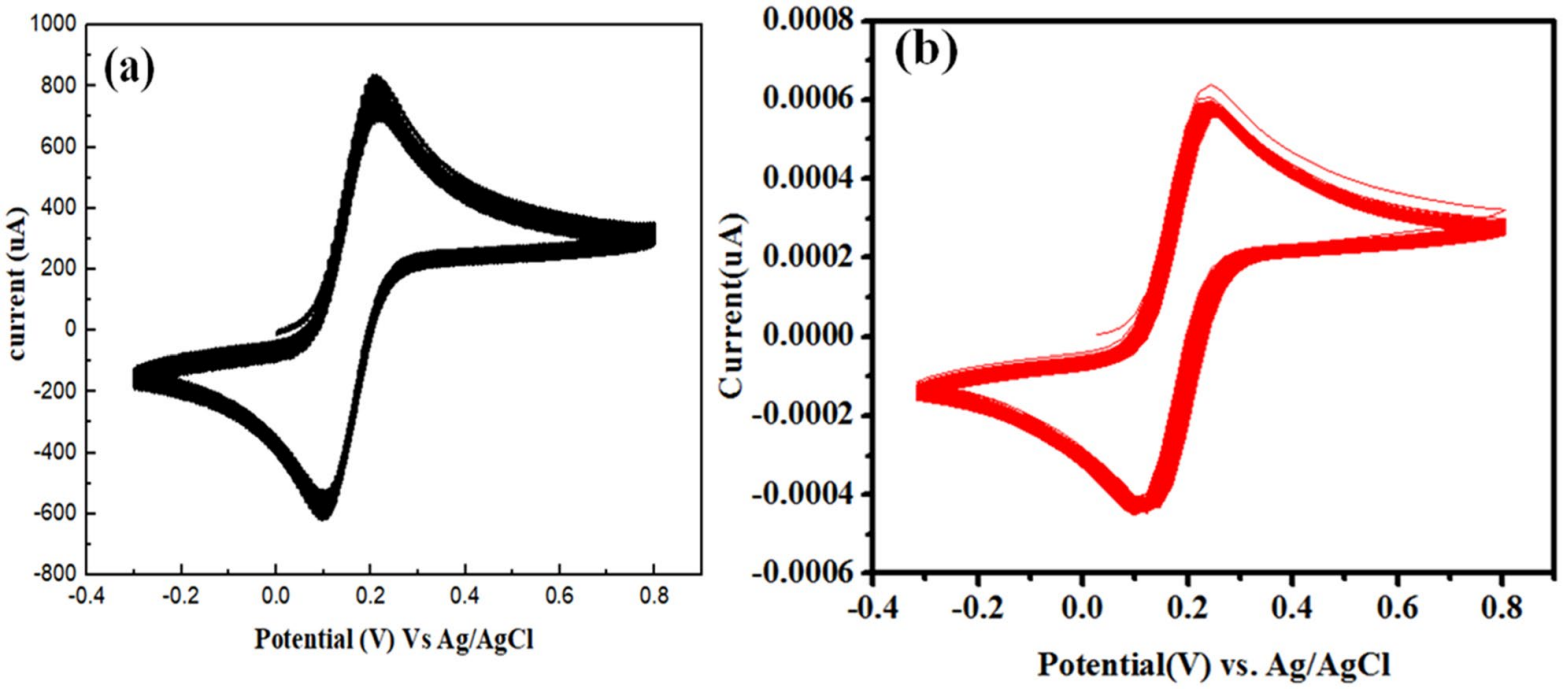
(**a**) Cyclic voltammogram of hydrogen peroxide with Te@ZIF-8-GCE at 100 cycles and (**b**) 1000 cycles.

The stability of Te@ZIF-8-GCE is tested by setting Te@ZIF-8-GCE of cyclic voltammetry by using 0.2 mM hydrogen peroxide solution in 0.1 M PBS of pH 7 (Fig. 4A,B). After passing by hundred to thousand cycles, both oxidation and reduction peaks are obtained at the same potential region as for the first cycle. The electrode material can thus reproducibly be re-used multiple times without compromising the efficiency.

### Amperometric analysis

**Figure 5:**
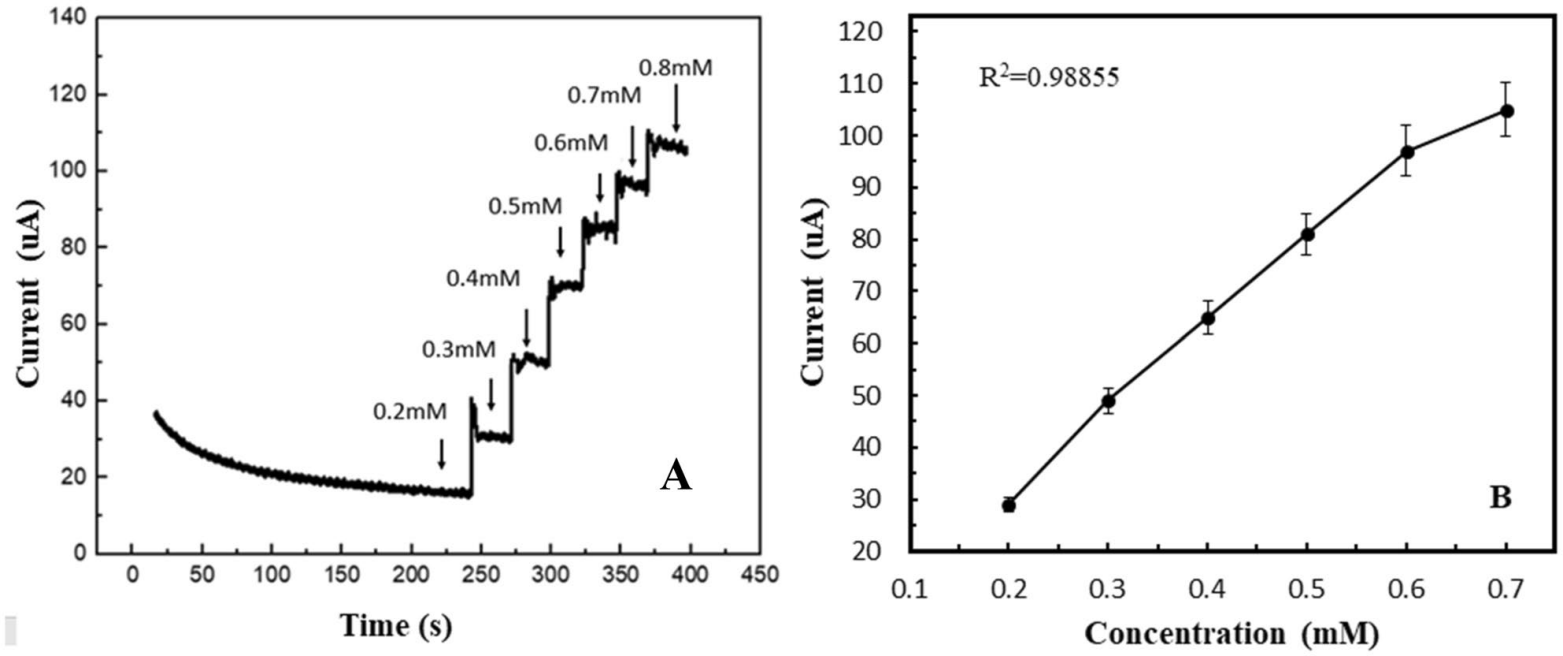
(**A**) Amperometric response of Te@ZIF-8-GCE upon successive addition of hydrogen peroxide in 0.1 M PBS of pH 7 at potential − 0.5 V to + 0.5 V. (**B**) Corresponding linear graph.

Amperometry is effective for checking the effect of change in analyte concentration at Te@ZIF-8-GCE. Figure 5A shows an increase in current response with the increase of concentration from 0.2 to 0.8 mM of hydrogen peroxide under the potential of − 0.5 V to + 0.5 V at pH 7 in 0.1 M PBS. The oxidation current density increases by the subsequent addition of hydrogen peroxide and attains a steady state. The calibration curve plotted for current against hydrogen peroxide concentration shows linear relation with R^2^ = 0.98855 (Fig. 5B). The limit of detection for hydrogen peroxide is calculated as 60 µM.

### Quantitative determination of hydrogen peroxide from serum samples of pancreatic cancer patients

The recovery of hydrogen peroxide is evaluated on Te@ZIF-8-GCE by spiking the known concentrations of hydrogen peroxide in human serum of pancreatic cancer patients, diagnosed with CA 19-9. The serum is diluted twenty times with a PBS solution of pH 7. The amperometric response of hydrogen peroxide is measured in the serum by the standard addition method. The detection results are listed in Table 1 where recoveries from serum samples are 93% to 101.6%. Variation in recoveries is due to the varying concentrations of hydrogen peroxide in serum samples.

**Table 1:** Recoveries of hydrogen peroxide from human serum by Te@ZIF-8-GCE.

Samples	Added conc. (mM)	Found conc. (mM)	Recovery (%)
1	1	0.93	93
2	5	4.81	96.2
3	10	9.69	96.9
4	15	14.77	98.5
5	20	19.93	99.6
6	25	25.11	100.3
7	30	30.36	101.6

Finally, Te@ZIF-8-GCE is used for the quantitative determination of hydrogen peroxide from serum samples of pancreatic cancer patients (Fig. S3). Seven serum samples are tested by the Elecsys CA 19-9 Assay to determine CA 19-9 concentration (Table 2). The concentration of hydrogen peroxide is then checked by Te@ZIF-8-GCE under optimized conditions. Current values for the samples indicate serum hydrogen peroxide levels where current increases with the increase of CA 19-9 concentration in serum (Table 2). A maximum current is obtained for the serum having a CA 19-9 level of 189 units mL^−1^. Findings suggest that the increasing levels of CA 19-9 in pancreatic cancer patients result in higher hydrogen peroxide production and can be electrochemically sensed using Te@ZIF-8-GCE.

**Table 2:** Levels of CA 19-9 (obtained from Elecsys CA 19-9 Assay) and hydrogen peroxide (calculated after sensing by Te@ZIF-8-GCE) from serum samples of pancreatic cancer patients.

No.	CA 19-9 (units mL^−1^)	Current (µA)	Conc. (µM)	Scan rate (mV/s)	Potential (V)
1	3.6	1.05	0.15	0.01	− 0.4 to + 0.8
2	30.0	2.66	0.39	0.01	− 0.4 to + 0.8
3	35.5	7.1	1.04	0.01	− 0.4 to + 0.8
4	67.37	9.8	1.44	0.01	− 0.4 to + 0.8
5	91.2	11.68	1.71	0.01	− 0.4 to + 0.8
6	92.6	16.01	2.35	0.01	− 0.4 to + 0.8
7	189	18.20	2.67	0.01	− 0.4 to + 0.8

## Conclusion

The tellurium doped zinc imidazole metal–organic framework (Te@ZIF-8) is prepared by the hydrothermal method. After a series of characterizations, the material is applied for hydrogen peroxide sensing. Parameters like scan rate, pH, and analyte concentration are optimized. Interference study is made in the presence of ascorbic acid and dopamine. Te@ZIF-8 shows stability up to 1000 cycles without significant change in electrochemical response. Impedance results confirm material roughness and porosity. Amperometry studies the effect of concentration via the standard addition method where current and concentration are linearly correlated. hydrogen peroxide recoveries from human serum samples range from 93 to 101%, depending on CA 19-9 levels in the samples. Material is also applied for the quantitative determination of hydrogen peroxide in pancreatic cancer samples, previously diagnosed with CA 19-9. Results demonstrate Te@ZIF-8 as a promising choice for hydrogen peroxide detection and can also be used for the detection of other reactive oxygen species.

## Supplementary information

Supplementary Information.
