# Supplementary material for: Tellurium doped zinc imidazole framework (Te@ZIF-8) for quantitative determination of hydrogen peroxide from serum of pancreatic cancer patients
Source: Sci Rep. 2020 Dec 3;10:21077. doi: 10.1038/s41598-020-78115-6 (PMC7713350; doi:10.1038/s41598-020-78115-6)
Supplement: Supplementary file 1 — Supplementary Information. [file 41598_2020_78115_MOESM1_ESM.docx]

**Tellurium Doped Zinc Imidazole Framework (Te@ZIF-8) for Quantitative Determination of Hydrogen Peroxide from Serum of Pancreatic Cancer Patients**

Batool Fatima^1*^, Dilshad Hussain^2^, Adeela Saeed^3^, Muhammad Salman Sajid^4^, Saadat Majeed^4^, Rahat Nawaz^4^, Muhammad Naeem Ashiq^4^, Muhammad Najam-ul-Haq^4*^, Rubaida Mehmood^5^

^1^ Department of Biochemistry, Bahauddin Zakariya University, Multan 60800, Pakistan

^2^ HEJ Research Institute of Chemistry, International Centre for Chemical and Biological Sciences, University of Karachi, Karachi (75270), Pakistan.

^3^ Department of Chemistry, The Women University Multan, Pakistan.

^4^ Institute of Chemical Sciences, Bahauddin Zakariya University, Multan 60800, Pakistan

^5^ MINAR Cancer Hospital, Pakistan Atomic Energy Commission, Pakistan

* Corresponding Authors

**Prof. Dr. M. Najam-ul-Haq**

Institute of Chemical Sciences

Bahauddin Zakariya University

Multan 60800 Pakistan

Tel.: +92 306 7552653

Email: [najamulhaq@bzu.edu.pk](mailto:%20najamulhaq@bzu.edu.pk%20)

**Dr. Batool Fatima**

Department of Biochemistry

Bahauddin Zakariya University

Multan 60800 Pakistan

Email: batoolfatima@bzu.edu.pk


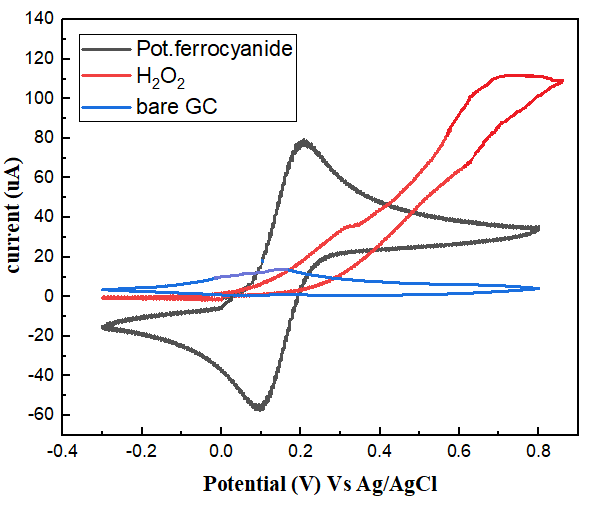


**Fig S1.** Cyclic voltammetric behavior of potassium ferrocyanide at bare glassy carbon electrode and at Te@ZIF-8-GCE. Electrochemical behavior of H_2_O_2_ at Te@ZIF-8-GCE.


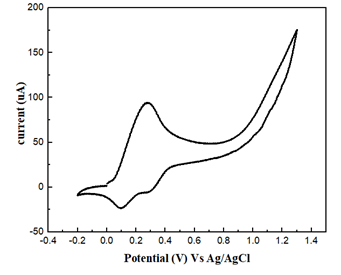


R^2^=93637

**Fig S2.** Cyclic voltammogram showing redox behavior of H_2_O_2_ in the presence of interfering species (ascorbic acid and dopamine) at Te@ZIF-8-GCE in 0.1 M PBS of pH 7. Conditions: potential window -0.4 V to +1.4 V, Scan rate 0.01 mV/s.


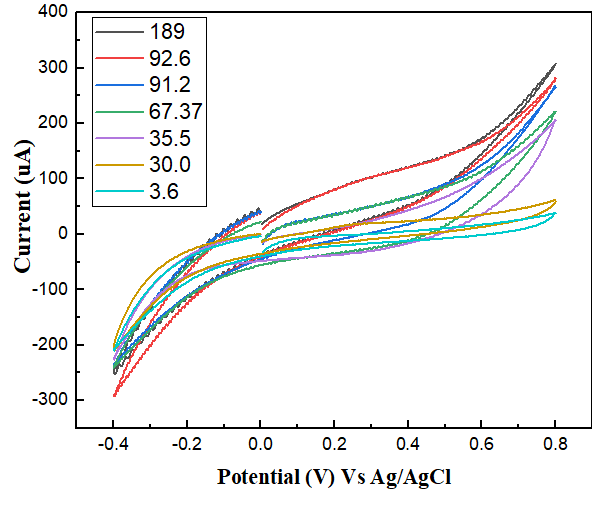


**Fig S3.** Cyclic voltammogram showing redox behavior of H_2_O_2_ in serum of pancreatic cancer samples at Te@ZIF-8-GCE. Conditions: potential window -0.4 V to +0.8 V, Scan rate 0.01 mV/s.
